# Methodological and reporting recommendations for clinical trials in Nutritional Psychiatry: Guidelines from the International Society for Nutritional Psychiatry Research

**DOI:** 10.1017/S0007114524001946

**Published:** 2026-03-28

**Authors:** Wolfgang Marx, Marjolein Visser, Caroline Wallace, Felice N. Jacka, Jessica Bayes, Heather Francis, Rachelle Opie, Meghan Hockey, Scott B. Teasdale, Almudena Sanchez Villegas, Adrienne O’Neil, Kuan-Pin Su, Julia J. Rucklidge, Michael Berk, Adrian Lopresti, David Mischoulon, Jeanette M. Johnstone, Heidi M. Staudacher

**Affiliations:** 1 Deakin University, Institute for Mental and Physical Health and Clinical Translation (IMPACT), Food & Mood Centre, School of Medicine, Geelong, VIC, Australia; 2 Department of Health Sciences, Faculty of Science, Vrije Universiteit Amsterdam, Amsterdam, Netherlands; 3 Amsterdam Public Health Research Institute, Amsterdam, the Netherlands; 4 School of Nutrition Sciences, University of Ottawa, Ottawa, ON, Canada; 5 Faculty of Health, Southern Cross University, East Lismore, NSW, Australia; 6 School of Psychological Sciences, Macquarie University, Sydney, Australia; 7 Neurology Department, Royal North Shore Hospital, Sydney, Australia; 8 Food for Thought Nutrition and Dietetics, Burwood, VIC, Australia; 9 Discipline of Psychiatry and Mental Health, School of Clinical Medicine, UNSW Sydney, Kensington, NSW, Australia; 10 Biomedical Research Center Network on Physiopathology of Obesity and Nutrition (CIBEROBN), Institute of Health Carlos III, Madrid, Spain; 11 ISFOOD – Institute for Innovation & Sustainable Development in Food Chain, Universidad Pública de Navarra (UPNA), Instituto de Investigación Sanitaria de Navarra (IdiSNA), Pamplona, Spain; 12 Mind-Body Interface Research Center (MBI-Lab), China Medical University Hospital, Taichung, Taiwan; 13 An-Nan Hospital, China Medical University, Tainan, Taiwan; 14 School of Psychology, Speech and Hearing, University of Canterbury, Christchurch, New Zealand; 15 Clinical Research Australia, Perth, WA, Australia; 16 Department of Psychiatry, Massachusetts General Hospital, Harvard Medical School, Boston, MA, USA; 17 Oregon Health & Science University, Center for Mental Health Innovation, Department of Psychiatry, Portland, Oregon, USA

**Keywords:** Guidelines, Nutritional psychiatry, Clinical trials, Mental health

## Abstract

Research on nutraceutical and dietary interventions in psychiatry has grown substantially, but progress is hindered by methodological inconsistencies and limited reporting standards. To address this, the International Society for Nutritional Psychiatry Research presents the first guidelines on clinical trial design, conduct, and reporting for future clinical trials in this area. Recommendations were developed using a Delphi process including eighteen researchers with considerable clinical trial expertise and experience in either methodology, nutraceutical, or dietary interventions in psychiatry. These guidelines provide forty-nine recommendations for clinical trial design and outcomes, five for trial reporting, and seven for future research priorities. The recommendations included in these guidelines are designed to inform both nutraceutical and dietary clinical trial interventions in Nutritional Psychiatry. Common themes include an emphasis on the importance of a multidisciplinary research team and integration of co-design processes into the conduct and design of clinical research, methods to improve transparency and replicability of trial outcomes, and measures to address common biases in nutrition trials. Furthermore, we provide recommendations for future research including examining a greater variety of nutraceutical and dietary interventions, scalable delivery models, effectiveness and implementation studies, and the need to investigate these interventions in the prevention and management of less studied psychiatric conditions (e.g. schizophrenia and bipolar disorder). Recommendations included within these guidelines are intended to improve the rigor and clinical relevance of ongoing and future clinical trials in Nutritional Psychiatry.

Over the last decade, there has been substantial growth in research into the role of nutraceutical and dietary interventions in psychiatry^(1)^. Observational studies in both adults and children have identified a broad range of nutrient-dense dietary patterns that are inversely associated with several psychiatric disorders, including anxiety, Attention-deficit/hyperactivity disorder (ADHD), and particularly depression^(2–5)^. The converse has also been demonstrated for Western dietary patterns^(2,6)^. Furthermore, crucial to informing our understanding of the efficacy of nutraceutical and dietary interventions are a growing number of published randomised controlled trials in Nutritional Psychiatry. Meta-analytic studies of clinical trials provide varying levels of support for nutraceutical interventions across a broad range of psychiatric conditions^(7–9)^. For example, a meta-review of thirty-three meta-analyses (*n* 10 951 individuals) found highly variable evidence for specific individual nutraceuticals in a given disorder and high variability in evidence for specific nutraceutical compounds across a range of psychiatric conditions^(7)^. There are also an increasing number of randomised controlled trials that provide evidence for the use of dietary interventions in psychiatry for both adults and children^(10,11)^. These include the Mediterranean diet for people with depression^(11–15)^, or elimination diets for people with ADHD^(16)^. Emerging trials have also investigated dietary interventions in other psychiatric disorders such as autism^(17)^ and bipolar disorder^(18)^. In response to this developing evidence, recent clinical guidelines have been published by the International Society for Nutritional Psychiatry Research and the World Federation of Societies of Biological Psychiatry^(19–21)^, which aim to improve translation of this evidence into practice.

However, recent guidelines have also cited methodological limitations such as small sample sizes, issues with fidelity or transparency of the composition of intervention, limitations with control conditions and blinding, and inconsistent reporting of key trial details as being common within the current literature^(21)^. For example, a recent systematic review and network meta-analysis of nutraceuticals in schizophrenia reported an average sample size of *n* 47 across the fifty studies included^(22)^. Similarly small sample sizes have also been reported in dietary interventions^(23)^. Such limitations can affect the reliability, validity, reproducibility, and generalisability of the trial results and their translation into real-world settings. There are also various areas of research that are currently understudied. For example, while there are several dietary intervention trials conducted in people with depression and in people with ADHD, evidence for other psychiatric conditions is sparse^(10,11,24,25)^. In schizophrenia, for example, most research on diet has focused on addressing metabolic syndrome rather than core psychiatric symptoms of the disorder^(24)^. This selective focus has led to a gap in our understanding and overlooks the potential benefits or risks of dietary interventions in less explored conditions. Furthermore, for psychiatric disorders such as major depressive disorder, several dietary interventions have focused on the Mediterranean diet whereas other diets (e.g. a low carbohydrate diet) have not been evaluated in controlled trials^(23,26)^. Studying other dietary approaches might unveil further effective strategies for psychiatric conditions. These gaps must be addressed in future clinical trials. Hence, the aim of these guidelines from the International Society for Nutritional Psychiatry Research is to provide guidance to clinical trial investigators for improving the quality and uniformity of clinical trial methodology, conduct, and reporting in Nutritional Psychiatry. These guidelines are designed to complement and be used in conjunction with other established nutrition-specific^(27)^, general reporting (e.g. CONSORT), ethical, and trial conduct guidelines (e.g.^(28–31)^).

## Methods

### Definitions

For these guidelines, we define whole diet interventions as any dietary intervention that aims to modify the intake of multiple food groups (e.g. Mediterranean diet, high fibre diet, and ketogenic diet). In contrast, food supplementation interventions focus on modifying the intake of a specific food item (e.g. consuming a fermented dairy product). We also use the term ‘nutraceutical’ to refer to interventions that consist of nutrients, compounds or microorganisms that are typically present in food and are delivered as a capsule or powder formulation.

### Scope and target audience

The recommendations included in these guidelines are designed to inform both nutraceutical and dietary (e.g. whole diet, food supplementation) clinical trial interventions in Nutritional Psychiatry. The recommendations are broad, aiming to improve quality across a diverse range of mental health conditions and methods. As the Nutritional Psychiatry evidence-base advances, more specific guidelines tailored to particular conditions, interventions or methods may provide targeted recommendations. While some included recommendations are applicable to many types of intervention studies (rather than Nutritional Psychiatry specifically), they are included due to the inconsistent application of these recommendations within the current Nutritional Psychiatry clinical trial literature. For example, the inclusion of people with lived experience in the design of trials is recommended by several peak bodies (e.g.^(32,33)^); however, we were unable to find any prior studies included in a relevant systematic review that described inclusion of this expertise in the trial development^(11)^. We acknowledge that not all recommendations will be appropriate for all trial designs and settings. Instead, rather than being a prescriptive set of guidelines, it is ultimately the purview of the investigator team to determine the suitability of the following recommendations in addressing the aims and research question of the individual trial. Similarly, most recommendations included in these guidelines are aimed at trials using efficacy study designs rather than effectiveness designs as this is how most of Nutritional Psychiatry research is currently being conducted, as indicated by recent systematic and scoping reviews in the field^(24,25)^. These guidelines are also aimed at informing investigator-initiated trials in Nutritional Psychiatry but may also be informative for industry sponsored trials. Finally, in recognition of the rapidly evolving research in the field of Nutritional Psychiatry, these guidelines should not be considered static; they are intended to be subject to regular reviews and updates as the field develops.

### Guideline development process

**Figure 1.**
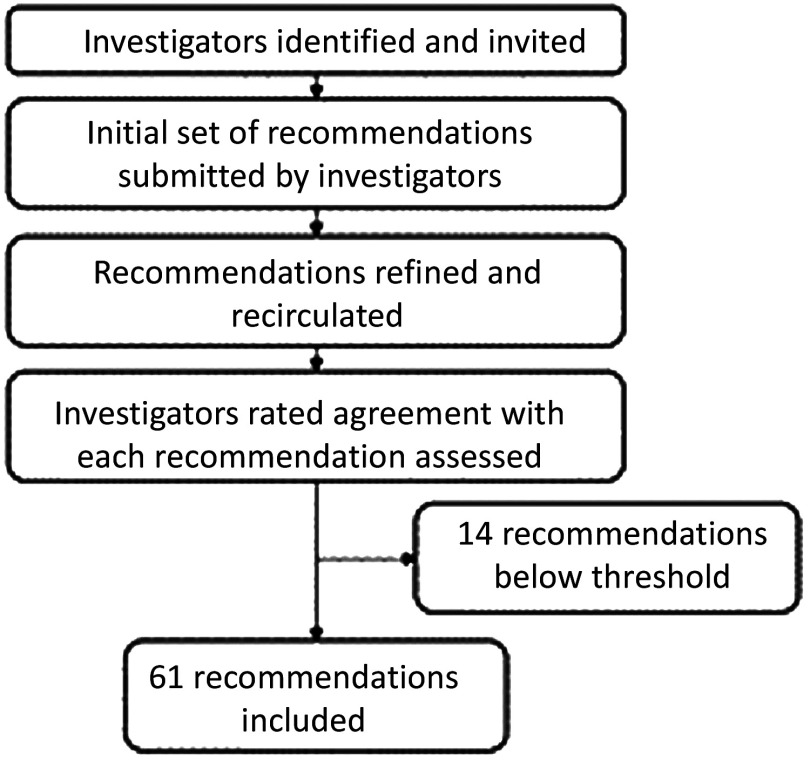
Figure 1 long description. Guidelines development process.

Using a similar approach to prior ISNPR guidelines^(34)^, recommendations were developed using a Delphi process (Fig. 1). In August 2023, the two lead authors (WM and HMS) established a working group of eighteen researchers with considerable clinical trial expertise and experience in either nutraceutical or dietary interventions in psychiatry. Investigators were primarily identified via reviewing members of relevant working groups and committees within the ISNPR and by reviewing previously published papers in Nutritional Psychiatry.

To generate an initial series of recommendations, two authors (WM and HMS) developed a questionnaire that asked the working group to provide recommendations pertaining to the following five categories: trial design considerations, participants, intervention, comparator, outcome, as well as any additional miscellaneous recommendations that did not fit these categories (online Supplementary Material). This questionnaire was designed to be open ended to capture a broad range of possible recommendations based on the individual researchers’ previous experience with clinical trials.

These submissions were then collated and refined by the lead authors to remove duplicate recommendations and to ensure consistent phrasing. From this, a ten-point Likert questionnaire was developed using REDcap^(35,36)^, whereby investigators were then asked to rate each recommendation from the refined list based on their level of agreement (0 – strongly disagree, 10 – strongly agree) or select ‘No answer’ if the specific recommendation was outside of their expertise. Recommendations that received an average of equal to or greater than 7 were included in the final manuscript, where the phrasing was further refined to improve clarity and uniformity. All excluded recommendations are available in the online Supplementary Material.

While this approach was designed to ensure a wide range of recommendations and to avoid potential bias caused by leading questions, we do acknowledge that such bias may still persist. Furthermore, consolidating similar responses into a single statement by the two lead authors (WM and HMS) may have introduced bias and possible misinterpretation of original statements.

## Clinical trial recommendations

Following the described process, we established forty-nine recommendations related to clinical trial design and outcomes (i.e. trial team, trial design, participants, intervention, comparator and outcome), five recommendations related to trial reporting and seven related to future research priorities. Here, we present all recommendations grouped by a specific methodological aspect and provide a discussion and summary of the background literature for the identified major themes.

### Trial team-related recommendations

#### Team composition

Ensuring relevant expertise and stakeholders are involved in trial design and conduct is likely to improve the rigor, feasibility, and clinical relevance of trial outcomes (Table 1). Dietitians accredited/registered with national governing bodies are particularly important to treatment delivery of whole diet interventions. Preliminary sub-group analyses of trials in psychiatric populations suggest dietitian-delivered interventions may provide a greater effect sizes in both depressive symptoms and metabolic outcomes^(11,37)^. This is understandable given the specialised skills needed to assess and provide dietary counselling to individuals, e.g. for addressing barriers and ambivalence to change, supporting optimal dietary adherence, and managing common co-morbid conditions such as disordered eating. This is particularly likely when the dietitian has experience and training within the mental healthcare setting. They are also best placed to collect and verify dietary data, as supported by a recent study demonstrating that 93 % of participant-completed diet records contained errors prior to dietitian verification^(38)^.

**Table 1.** Trial team-related recommendation statements Table 1 long description.

	Mean	sd	% rated ≥ 7·0
1. Teams should comprise investigators proficient in all relevant trial aspects, such as trial design, statistics, and clinical practice, and should comprise ethnic and gender diversity.	8·56	1·26	94
2. Engage individuals with lived experience of mental health disorders throughout all phases of the trial.	7·56	2·61	72
3. Dietary interventions should be delivered by Accredited Practising Dietitians/Registered Dietitians for best practice, rather than other clinicians or general research staff.	7·17	3·08	61

A further important consideration is the inclusion of stakeholder and consumer input in all phases of the trial, from conception and design through to dissemination. For intervention studies, this may involve people with lived experience of the investigated mental disorder whereas prevention studies may also include individuals from the general community. The inclusion of such expertise, via co-design or working groups, improves the feasibility and value of the intervention to participants and people with mental disorders. Because of these improvements, inclusion of lived experience is also increasingly expected from the general community, as well as the scientific community (e.g. major funding bodies, journals). Recently developed frameworks such as The Involvement Matrix^(39)^ aim to support participant involvement in research projects and can be used both prospectively to discuss the possible roles of stakeholders in different phases of projects and retrospectively to reflect on whether those roles were carried out satisfactorily.

### Trial design-related recommendations

#### Participant burden

Prior literature and consumer feedback notes the negative effects of substantial participant burden on recruitment and participant adherence^(40)^. Hence, the investigator team should carefully consider the quantity of and methods by which data are collected from participants in relation to the level of burden this poses to them (Table 2). This can be achieved through consultation with people with lived experience, qualitative workshops and feedback from prior participants, avoiding recruitment during periods where adherence is more difficult (e.g. major holidays; recommendation #8), careful consideration of instruments and the order in which they are administered to assess eligibility or to capture participant data, and thorough pilot testing and subsequent refinement of the trial protocol. Moreover, chosen outcome measures should take into consideration symptom presentations and cognitive impairments prevalent in specific psychiatric populations (e.g. cognitive dysfunction, anhedonia) that may affect a participant’s ability and willingness to reliably complete the outcome measures.

**Table 2.** Trial design-related recommendation statements Table 2 long description.

	Mean	sd	% rated ≥ 7·0
1. During intervention design, account for participant burden, resource constraints, and potential adherence barriers.	9·22	1·03	100
2. Clearly report treatment adherence assessment methods and outcomes and predefine adherence thresholds before commencing recruitment.	9·00	0·94	100
3. When collecting dietary data, implement strategies, such as food models and cross-checking with participants, to enhance accuracy of data.	8·22	1·58	83
4. Ensure trials are adequately powered with sufficient participant numbers to enable detection of between-group differences for the primary outcome.	9·33	1·05	100
5. Address and mitigate expectancy bias through strategies such as presenting all hypotheses as equally of interest and when ethically permissible, limiting participant exposure to intervention details, and measuring participant expectations in advance.	8·50	1·42	94
6. Studies should be blinded to the greatest extent possible. This includes measures to blind participants as well as research staff (e.g. personnel conducting assessments, principal investigators, and trial statisticians).	9·06	1·22	94
7. Design trials of sufficient duration for treatment to take effect and to capture both mid and long-term outcomes.	8·50	1·34	94
8. Account for seasonal variations, such as holiday breaks, during recruitment and data collection to avoid potential reduction in adherence.	7·89	1·76	67

#### Participant adherence

All trials should define intervention adherence *a priori* and then use that definition to measure adherence to the intervention to enable reporting and analysis of outcomes in the context of participant adherence. For example, previous trials have demonstrated a greater treatment response in participants who had a larger level of adherence compared with those with a lower level of adherence^(12,41)^. Tracking adherence trajectories throughout the duration of the trial may also be useful, particularly for refining processes that may be contributing to low adherence. There are several methods to assess adherence, and all have limitations that need to be considered by the trial investigators. Biological methods (e.g. blood levels of a nutrient in a nutrient supplementation trial) may offer an objective marker of intervention adherence but are more invasive than other methods. Self-reported methods (e.g. diet records) are inexpensive but are inherently prone to error and require additional dietetic time for participant training and record verification. Furthermore, researcher-reported endpoints (e.g. group session attendance) can be consistently measured by research staff but may be limited in precision, particularly in dietary interventions. For example, recording attendance to intervention sessions as a marker of adherence may not capture data related to participants leaving early or low levels of engagement.

#### Blinding and expectancy bias

An important issue raised in previous publications is the challenge of participant expectancy – where the expectations or beliefs of the participants contribute to their treatment response. This may be particularly pertinent to nutrition interventions compared with pharmaceutical interventions as individuals may hold strong beliefs about food and diet^(42)^. Whole diet interventions are also exceedingly difficult to blind, increasing the risk of unequal expectancy across groups. Broadly, expectancy can have implications for retention, as participants wanting to be assigned to the treatment group may drop out if they believe they have been assigned to the control group. Trial findings may also be affected as participants knowingly assigned to the control group may experience poorer mental health outcomes and/or participants knowingly assigned to the treatment may experience amplified mental health outcomes, both leading to overestimation of true treatment effects. Where ethically possible, designing advertising and participant information and consent materials to conceal the primary research hypothesis may help mitigate expectancy bias. Other trial design methods such as placebo run-in phases have also been proposed to reduce the number of placebo responders and enhance the chance of detecting a treatment effect; however, large meta-analyses of these study designs in the antidepressant literature have not been supportive^(43)^. Whilst methodologically challenging and resource-intensive, whole diet interventions should ideally be conducted with a placebo diet control. This is addressed further in section 3·5.

#### Follow-up duration

There is currently insufficient evidence to recommend a specific minimum duration for nutraceutical or dietary trials in Nutritional Psychiatry. Many recent dietary intervention trials have reported clinical effects in depression over a 12-week timeframe^(12,13,15)^; however, improvements have also been reported in as little as 3 weeks^(14)^. Nutraceutical intervention trials generally vary from 4 to 12 weeks. Prevention studies over one to 5-year periods have also been conducted in both dietary and nutraceutical interventions although have not reported improvements in onset or reoccurrence of depressive symptoms^(41,44,45)^. Determining trial duration should be guided by prior trials, mechanistic, and pharmacokinetic understanding of the bioactive components (particularly in the case of nutraceutical interventions) and feasibility considerations. A further consideration is that there are few intervention trials monitoring treatment effect beyond 6 months, which limits understanding of long-term feasibility, efficacy, and safety of interventions. This is important especially in the context of other fields of behavioural science (e.g. dietary interventions targeting weight loss for obesity) that show reduced adherence in longer intervention programs compared with dietary interventions of < 3 months.

### Participant-related recommendations

#### Recruitment practises and generalisability of trial population

Efficacy trials have historically been conducted in populations that are highly selected and seldom generalisable to the real-world clinical environment^(46)^. Key drivers of this are recruitment methods and intervention content that fail to reach or appeal to a broader population. To address this, efficacy trials should implement inclusive recruitment practices and strategies that promote diversity in relevant demographic factors (e.g. age, gender, race, ethnicity, socioeconomic status, and rurality; Table 3). This may involve leveraging community organisations and digital platforms to reach underrepresented groups. Incorporating flexible scheduling, teleconference visits, and/or participant travel reimbursement may improve accessibility and convenience of participating in trials and enhance participant diversity and representation. Furthermore, detailed reporting of demographic characteristics and *post hoc* analyses examining intervention effects across various subgroups can provide context to the generalisability of the findings to the wider target population.

**Table 3.** Participant-related recommendation statements Table 3 long description.

	Mean	sd	% rated ≥ 7·0
1. Implement recruitment and advertising strategies that minimise selection bias and enhance the generalisability of the sample to the broader population.	7·94	1·87	89
2. Account for comorbidities within eligibility criteria or document and interpret outcomes in the context of recorded comorbidities.	7·78	1·51	78
3. Establish clear protocols for managing participants with eating disorders and ensure broad and appropriate trial team expertise.	7·44	2·93	83
4. Consider baseline dietary practices in exclusion criteria (e.g. high baseline diet quality).	7·28	2·13	72
5. In mental health prevention interventions, prioritise high-risk participants to guarantee a sufficient number of cases during follow-up.	7·78	2·42	78
6. Focus on populations with distinct clinical needs and a high likelihood of treatment response. Avoid the potential floor effect by diversifying beyond exclusively healthy participants.	7·89	1·76	72
7. Use structured interviews and accepted cut-offs when formulating eligibility criteria related to mental disorder symptoms.	7·83	2·09	72

#### Consideration of comorbid conditions

There is a high prevalence of both physical and psychiatric comorbidity in people with mental disorders^(47)^. For example, one-third of total nonfatal burden in people with depression is estimated to be due to comorbid physical diseases and other studies report that people with depression have a substantially increased risk of eating disorders, as well as other psychiatric comorbidities, compared to the general population^(47,48)^. This high prevalence of comorbidity may affect treatment outcomes by introducing safety risks via increased drug–nutrient interactions, altering implicated mechanisms of action (e.g. Crohn’s disease altering the gut microbiota), and increasing risk of safety concerns (e.g. lipid profiles in people with comorbid coronary heart disease). Investigators should consider how to identify, manage, and examine treatment efficacy and safety in participants with a potential vulnerability to having an existing condition triggered by their participation such as eating disorders and disordered eating. Identification during the screening process is important for dietary interventions, and particularly those that involve any type of dietary restriction or food elimination, as these may trigger or exacerbate disordered eating behaviours. It is common practice in efficacy trials to exclude participants with common comorbidities to improve the homogeneity of the participant sample; however, this has also been criticised for reducing external validity and generalisability of the trials results^(49)^. Another approach is to employ an effectiveness design including ‘all-comers’ with an aim to reflect ‘real world’ clinical presentations. However, some comorbid conditions should still be considered for exclusion based on safety reasons (e.g. participants with eating disorders should not be eligible for whole diet trials requiring dietary restriction).

#### Consideration of ceiling and floor effects

Investigators should consider both potential floor and ceiling effects when selecting trial participant eligibility criteria. The floor effect, whereby participants exhibit low baseline scores in the variable of interest (e.g. baseline depressive symptoms), and therefore, only small improvements are possible, reduces the likelihood of separation between the intervention and control condition. For example, investigating an intervention in a participant sample in which baseline depressive symptoms are already low may impair the likelihood of seeing a treatment effect, particularly without a large sample size. Low baseline levels of depressive symptoms were cited as a possible explanation for the fewer than expected number of transitions to Major Depressive Disorder (MDD) in the large MoodFOOD prevention trial (10 % after 12 months *v*. an expected 33 % based on previous studies)^(1,41)^. Similarly, consideration of a possible ceiling effect should be applied when selecting the eligibility criteria for a trial. For example, high baseline diet quality of participants could lead to a ceiling effect in trials evaluating the effect of a high diet quality whole diet intervention on depressive symptoms. Several prior dietary intervention trials in this field have included diet quality screening tools as part of their eligibility criteria to mitigate this issue^(12–15)^.

### Intervention-Related Recommendations

#### Research question-informed trial design

Investigators should ensure that the selected study design is best suited to address the intended research question (Table 4). This is particularly relevant to dietary intervention trials where there are a variety of possible delivery options (e.g. dietary counselling, controlled feeding trials, or their combination) and where there are potentially other aspects of the intervention that may influence treatment outcomes. For example, motivational interviewing and other commonly used behavioural components may independently influence mental health outcomes by improving feelings of mastery, behavioural activation, improved locus of control, and self-efficacy. Therefore, if the trial is intended to investigate the therapeutic value and/or biological mechanisms of a whole diet intervention, a controlled feeding trial may be preferable. In these trials, most/all food is provided and dietary counselling is not necessary. Although costly and intensive, these trials also have the added benefit of being amenable to double blinding and facilitating strict adherence. Collinearity is another complexity that should be considered in interpretation of whole diet and food supplementation trials. This is the phenomenon in which increasing or decreasing intake of foods or food groups can influence background dietary intake that in itself could influence outcomes. Similarly, the choice of delivering the intervention as a stand-alone intervention, adjunct to psycho- or pharmaco- therapy, or multicomponent should be guided by the intended research question, the qualifications, and credentials of the lead investigator and should consider how results of the trial could feasibly be integrated into clinical practice. Formal tools such as the PRECIS-2 tool may be used to ensure the trial design and decisions are in line with the intended purpose of the trial^(50)^.

**Table 4.** Intervention recommendation statements Table 4 long description.

	Mean	sd	% rated ≥ 7·0
1. Investigators should determine if the intervention will be investigated as a monotherapy, adjunct to standard care, or in combination with other treatments (e.g. diet-nutraceutical, diet-exercise).	8·39	1·89	78
2. Manualise whole diet or behavioural interventions, conducting fidelity testing to ensure exposure integrity.	8·06	1·18	100
3. Ensure that dietary interventions involve research dietitians or clinical nutritionists proficient in relevant counselling techniques, like motivational interviewing, to bolster intervention adherence.	8·67	1·70	89
4. For dietary interventions, include comprehensive guidelines on food safety, hygiene, storage, and preparation, especially when introducing unfamiliar foods.	7·22	2·88	72 %
5. For specific participants (e.g. rural or remote areas or if high risk of food insecurity), ensure ingredient availability in food supplementation or whole diet interventions.	8·06	1·58	78
6. Ensure intervention delivery is tailored to the included population demographics (e.g. culture, age, gender) and accommodates common psychological symptoms like reduced motivation or attention.	8·72	1·63	83
7. Consider various modes of delivery for dietary trials, including dietary counselling, controlled feeding (in which most or all food is provided) and hybrid trials (counselling and feeding) based on the research question.	8·17	1·57	83
8. Consider the potentially independent influence of motivational counselling on mental health outcomes when designing and interpreting trials.	8·11	1·63	83
9. Focus dietary interventions on ad libitum or weight-neutral designs unless targeting weight loss specifically.	8·17	1·92	83
10. Decide between individual or combined nutraceutical compounds for interventions, providing a rationale for the chosen design.	7·33	2·45	67

#### Standardising dietary and nutraceutical interventions

Dietary interventions are complex interventions, and their design requires consideration of several factors to ensure effectiveness and replicability. First, in contrast to drug or nutraceutical interventions where there is generally a higher level of uniformity in how the intervention is administered, dietary interventions require additional procedures to ensure intervention fidelity, reproducibility, scalability and, ideally, nutritional adequacy for participants. These include processes such as manualising the intervention delivery procedure, additional and ongoing training of the trial staff that are delivering the intervention to ensure standardisation, and conducting fidelity testing and audits of intervention delivery. The degree of standardisation may also differ by trial design. Efficacy trials may seek a higher level of standardisation to ensure a highly precise and ‘optimal’ delivery of the intervention, whereas an effectiveness trial may deliver the intervention on a personalised basis depending on client characteristics and other clinical considerations and staff resources, to reflect real world delivery. Standardisation is also an important consideration of nutraceutical interventions, particularly for herbal or plant-based formulations, in which the nature and dose(s) of the bioactive component(s) may vary considerably^(51,52)^. This can be facilitated by independent testing for bioactive constituents as well as using formulations that standardise to a specific bioactive content.

#### Individualised and culturally applicable dietary interventions

Designing dietary interventions tailored to the circumstances of the target population is likely to result in greater appeal and participant adherence. This includes consideration for individual taste and cultural preferences. Demographic considerations such as age and gender should also be considered. For example, in relation to a recent Mediterranean diet Randomised Controlled Trial (RCT) in young men with depression, Bayes *et al.* ^(53)^ highlighted the need to consider pervasive gendered associations with specific foods (e.g. meat perceived as ‘masculine’, vegetables perceived as ‘feminine’) and how this may influence diet adherence in differing trial populations. Other relevant considerations include the cost and availability of the investigated dietary intervention, food preparation skills, available time, geographical access to foods, and treatment duration. Socio-cultural factors such as economic circumstances and health literacy are also important. Previous qualitative studies of participants undergoing a Mediterranean dietary intervention highlighted the perceived increase in costs associated with adherence to the diet as well as barriers related to increased food preparation time and availability of some food items within their region^(54,55)^. To mitigate this, prior trials have provided the costliest components of the diet (e.g. extra virgin olive oil, nuts) to participants as part of a food hamper^(12,13)^. Similarly, we recommend education regarding food safety, hygiene, storage, and preparation in populations that may be unfamiliar with the intervention food items and practices. While also relevant to nutraceuticals, whole diet counselling interventions especially need to accommodate for common psychological symptoms associated with disease pathogenesis and/or medication side effects like changes in appetite, reduced cognition motivation, fatigue, and attention, which may substantially impair the ability to strictly adhere to intervention recommendations. The use of research dietitians proficient in counselling techniques is recommended to mitigate many of these potential barriers.

#### Weight-neutral dietary interventions

While weight loss may improve psychiatric outcomes such as depressive symptoms in people with obesity^(56)^, prior dietary interventions suggest that weight loss is not a requirement in Nutritional Psychiatry trials for a beneficial mental health outcome^(12)^. This evidence is largely based on trials conducted in MDD and requires further investigation in populations where metabolic dysfunction and overweight are prominent features (e.g. schizophrenia). However, based on the currently available evidence, for dietary intervention trials in which mental health is a primary outcome, a weight-neutral approach that avoids weight-centred language or motivations should be used. This approach has additional benefits. First, such strategies help to avoid potential safety issues including reduced body dissatisfaction, disordered eating patterns, and stigma that are counterproductive to participant health. Second, preliminary evidence from other lifestyle interventions in mental disorders suggests emphasising mental health benefits, rather than physical health benefits, of an intervention may improve adherence^(57–59)^. Finally, ensuring bodyweight stability avoids the challenge of dissecting whether improvement in outcomes has occurred in response to the intervention or from the contemporaneous weight loss. This is particularly important in efficacy trials.

#### Consideration for combination or individual nutraceutical formulations

The choice to investigate a nutraceutical intervention as an individual compound or a multi-compound formulation should be guided by the primary research question. Single nutrient interventions offer greater insight into the specific clinical effect and mechanisms of a specific nutrient; however, a multi-nutrient formulation may be more representative of dietary consumption, their actions *in vivo,* and possibly offers synergistic effect^(60,61)^.

### Comparator recommendations

#### Comparator selection

The gold standard trial design for assessing treatment efficacy in pharmaceutical trials is the placebo-controlled randomised controlled trial. The design of placebos in pharmaceutical trials is relatively straightforward; a pill can be designed to exactly match the treatment in appearance, taste, and even texture, whilst being ‘inert’ (i.e. inactive) and free of the drug under investigation, meaning participants can be blinded to their treatment allocation. In most cases, nutraceutical trials can also be tested in a blinded placebo-controlled fashion relatively easily as they usually investigate a treatment delivered as a pill or powder. Plant-derived nutraceutical interventions with distinct smells (e.g. ginger supplementation) are an exception to this and where the smell of the placebo condition requires consideration. Diet interventions, however, are exceedingly difficult to investigate in a placebo-controlled fashion as no foods or diets are ‘inert’ and cannot be easily blinded. In the case of food supplementation trials, in which an alternative food with the same energy content is provided in the control arm, the intent of the trial can be masked to mitigate the risk of unequal expectation bias across groups (Table 5). In the case of whole diet trials, placebo diets can be implemented but are challenging to design and deliver; however, as above, these should be considered the gold standard where resources allow^(62)^. Feeding trials, in which all food and fluid is provided to participants, can facilitate blinding and placebo control. Whole diet counselling trials are more difficult to placebo control, and as such, there has been no placebo-controlled trial of a whole diet counselling intervention to date. Instead, common control conditions that have been previously used in superiority trials include befriending protocols^(12)^. These are non-dietary approaches that can control for the level of engagement and support provided by being involved in a research study, although do not exactly match the motivational counselling that is often provided in dietary counselling trials. Recommendations for the design of placebo diets, such that engagement and blinding and all aspects of diet (e.g. shopping, cooking to follow a specific diet) other than the active component are equivalent to the intervention group, have been reviewed elsewhere^(62)^. Non-inferiority trial designs in which treatments are compared with currently accepted interventions (e.g. antidepressant medications, psychotherapy) are important once initial efficacy has been established^(63)^.

**Table 5.** Comparator recommendation statements Table 5 long description.

	Mean	sd	% rated ≥ 7·0
1. The choice of control condition should be clearly explained and justified.	9·28	0·99	100
2. Prioritise the use of placebo or best-practice comparators over waitlist controls.	7·56	2·59	72
3. In dietary interventions, when achieving effective blinding is challenging, attempt to match expectancy and engagement in the intervention and control.	8·50	1·61	89
4. For interventions involving nutraceuticals or foods, the placebo should be matched in appearance, taste, smell, and packaging.	7·83	2·19	83
5. Consider employing placebo diets and rigorously reviewing trial materials to sustain blinding.	7·72	2·76	83
6. Consider use of non-inferiority trial designs to compare Nutritional Psychiatry interventions to currently recommended treatments (e.g. cognitive behavioural therapy, pharmacotherapy).	7·67	2·60	78

### Outcome-related recommendations

#### Clinical outcome considerations

To enhance the accuracy and depth of outcome measurement, it is recommended that trials capture both self-report and clinician-rated assessments of the clinical outcome of interest, where possible (Table 6). This is particularly pertinent to trials where participants are unblinded, and self-reported outcomes may be more likely to be influenced by expectancy. As with all behavioural trials, a related important consideration is to avoid confirmation bias by ensuring clinician assessments are blinded and are not conducted by the intervention or unblinded research staff^(30)^. To help inform future research, we also recommend that sub-domains of the primary outcome (where they are validated) are analysed in addition to total scores to investigate the effect of the interventions on individual aspects of mental health. However, investigators should also be mindful that these exploratory analyses may be underpowered when interpreting the results. Further, it is advised to measure symptoms at multiple pre-specified timepoints. This may involve momentary time sampling methods, such as ecological momentary assessment, to track the progression and fluctuation of symptoms over time, providing a more detailed picture of the effect of the intervention by capturing temporal dynamics.

**Table 6.** Outcome-related recommendation statements Table 6 long description.

	Mean	sd	% rated ≥ 7·0
1. For clinical outcomes, employ both self-report and clinician-rated methods to optimise outcome accuracy.	8·39	1·46	89
2. Consider measuring clinical symptoms at multiple prespecified timepoints and/or through the inclusion of momentary time sampling.	8·33	1·41	89
3. Consider reporting both total scores and individual symptom changes from clinical assessment tools.	7·17	2·19	78
4. Consider relevance of assessing both psychiatric (e.g. depressive symptoms) and metabolic/physical (e.g. cholesterol) outcome measures.	7·83	1·46	83
5. Interventions should consistently measure and report potential confounders such as background diet composition, medical history, demographic data, and biomarkers, psychological and medical treatments at baseline and throughout the intervention period.	8·28	1·52	83
6. Consider analysing the treatment effect in relevant subgroups (e.g. depressive subtypes) and examining of moderators of treatment effect (e.g. age, sex).	7·89	1·20	89
7. Consider collecting biological samples (e.g. blood, stool) and ensure adequate statistical power for analysing potential mechanistic and treatment-response biomarkers.	8·17	1·17	94
8. Evaluate blinding success at trial conclusion using structured techniques for both participants and research teams.	7·50	2·36	78
9. Weigh the pros and cons of various validated dietary assessment methods and select based on the trial’s objective. Where evidence supports, incorporate validated dietary biomarkers as objective intake and adherence measures.	8·33	2·49	83
10. Ensure dietary assessments are either administered or verified by a dietitian to enhance accuracy.	7·89	2·23	72
11. Include economic analyses from health economists, when feasible, to evaluate cost-effectiveness.	8·17	1·01	100
12. Manuscripts should discuss trial outcomes within the broader context of trial-related burdens, associated costs, and clinical feasibility.	7·72	2·21	89
13. Assess both acceptability and tolerability of the intervention.	8·17	1·26	89
14. Safety assessments should be conducted periodically throughout the trial period.	8·28	2·08	89
15. Use assessment tools validated for the target population and contextualise them within established minimum clinical differences.	8·89	1·49	89

#### Assessment of both psychiatric and metabolic outcomes

While there are a growing number of individual trials that have investigated the efficacy of dietary and nutraceutical interventions for psychiatric outcomes and metabolic outcomes in psychiatric samples^(7,64)^, many trials, particularly for dietary interventions, have not examined both sets of outcomes within the same trial. Considering the shared mechanistic pathways that are implicated in both psychiatric and physical outcomes^(47)^ and the substantial time, cost, and effort required for running clinical trials, it is both prudent and informative to incorporate measures of both outcome types into Nutritional Psychiatry trials.

#### Dietary assessment considerations

It is recommended to weigh the pros and cons of various validated dietary assessment methods and select an approach based on the study’s objective and participant burden. For example, in nutraceutical interventions, a comparably brief measure that captures broad changes in diet (e.g. clinician-administered diet history) and/or changes in the specific nutrients under investigation might be preferable whereas for a dietary intervention, a more comprehensive dietary assessment such as a food record may be required. Where evidence is supportive, incorporate validated dietary biomarkers as objective intake and/or adherence measures. For example, a spectrophotometer has been used to evaluate plasma carotenoid levels to verify adherence to a healthy eating-Mediterranean diet intervention in young adults^(14)^. This has also been covered within the Participant Adherence section. Furthermore, due to the well-established limitations of self-reported dietary intake, we recommend that dietary assessments are administered and verified by a dietitian (e.g. cross-checking of food records with a participant) to ensure consistent and reliable data collection. This is particularly important in serious mental illness where challenges to recall (e.g. cognitive dysfunction) are common and where most dietary assessment tools have not been validated within this participant population.

#### Markers of treatment response

Trial investigators are encouraged to align with precision medicine principles^(65)^ and consider collecting comprehensive baseline data on biological, clinical, and demographic variables that may influence treatment response. These data can facilitate the identification of specific subgroups that are particularly amenable to treatment response. For example, prior trials have suggested elevated baseline inflammatory markers may influence treatment response to nutraceutical interventions^(66)^. Furthermore, a recent umbrella review identified a range of demographic factors that may positively (e.g. higher education status, female gender) or negatively (e.g. previous negative life events) influence treatment response to psychiatric treatments^(67)^. However, as per the conclusions of a recent systematic review that investigated this in Nutritional Psychiatry^(68)^, evidence is currently preliminary, and further trials are required to investigate other unexplored factors that may influence treatment response. An important consideration in implementing these analyses is to ensure that there is adequate statistical power to detect such factors as failure to do so can increase the risk of type 2 statistical error.

#### Blinding assessment

Trials should assess the efficacy of the implemented blinding processes. Due to the use of subjective assessment tools (e.g. self-reported depressive symptoms), this is particularly important for psychiatric assessment; however, recent evidence suggest that blinding is rarely assessed^(69)^. Quantitative data on blinding efficacy provide important context for interpretation of the results. Such assessment should be undertaken for all blinded parties. For example, in a double-blind intervention, blinding assessment for both participants and research staff will be informative. This is relevant to both nutraceutical and dietary interventions alike; however, this is particularly pertinent to dietary interventions due to the complexity of introducing effective blinding procedures and the higher risk of unblinding. There are various methods proposed for implementing blinding assessments. Many trials simply ask participants to guess their allocation (i.e. treatment, control and unknown) at the end of the trial. In these cases, equal responses across groups and/or between treatment and control groups would be indicative of successful blinding. Statistical methods are also available such as the James’ and Bang’s blinding indices^(70,71)^. Although a controversial topic, the timing of evaluating blinding success traditionally has been at the end of the trial, but it has been suggested that this may be more accurate if undertaken earlier in the trial before participants have hunches about intervention efficacy or even experience side effects as a result of receiving the intervention^(72)^. Furthermore, exploring the reasons for why participants believe they are in the intervention or control condition can be informative. For example, a higher number of participants correctly guessing they are in the active intervention may be due to a range of reasons including participants experiencing improvements in outcomes, distinct smell and presentation of the intervention (e.g. nutraceuticals), and adverse effects, all of which can influence the interpretation of blinding success.

#### Inform clinical translation efforts

To advance the clinical translation of trial findings, researchers are encouraged to incorporate outcomes such as cost-effectiveness and safety, acceptability, and tolerability. The latter can be assessed using validated tools and adverse reporting methods, qualitative methods such as structured clinical interviews, or implementation assessments such as through the use of the RE-AIM framework^(73)^. Furthermore, in addition to traditional effect size and cut-off metrics, the results should also be reported within the context of treatment costs, treatment burden, treatment-related adverse effects, and clinically meaningful differences to determine if the findings are not only statistically robust but also practically significant in a real-world setting.

### Reporting recommendations

#### Reporting considerations

To improve replication efforts in Nutritional Psychiatry, authors are encouraged to provide detailed descriptions of all details relevant to the delivery of the intervention under investigation (Table 7). Using reporting frameworks such as Tidier and CONSORT (and its relevant extensions) are strongly encouraged throughout design, conduct, and reporting^(28,29)^. Pre-registration of the trial with a national clinical trial registry prior to commencing recruitment is required. Publication of the trial protocol is recommended during recruitment and submission of the full study protocol submitted to the journal upon submission of results. Furthermore, as some journals have word restrictions that may prohibit detailed descriptions of all aspects of methodology and/or interventions, authors are encouraged – and indeed, this is a common requirement of many medical journals – to include these in supplementary information or to publish additional resources that provide further information such as separate publications (e.g.^(74)^) or stored on publicly accessible repositories such as pre-print archives (e.g. Open Science Framework).

**Table 7.** Reporting recommendation statements Table 7 long description.

	Mean	sd	% rated ≥ 7·0
1. Whole diet trials should describe both the treatment and control diets in detail, including relevant food groups and nutrient composition. Nutraceutical trials should detail the dose, frequency, composition, and timing of the intervention.	8·89	1·45	89
2. If dietary counselling is provided, investigators should report behavioural-change techniques and counselling styles employed, the mode of the delivery, supplementary resources provided, and the frequency and duration of sessions.	9·22	0·92	100
3. For industry-funded trials, clearly delineate the extent of industry involvement in the manuscript.	9·61	0·68	100
4. Authors must transparently declare conflicts of interest and all potential biases.	9·67	0·58	100
5. Trial protocols should be made publicly available, via published protocols or online repositories, to support replication and extension.	8·06	2·37	78

#### Funding and conflict of interest transparency

Due to the reported risk of bias associated with industry funding in medicine^(75)^, our guidelines provide strong endorsement for the need to clearly report industry funding and degree of involvement in all aspects of the clinical trial, data analysis and interpretation, and manuscript development. Resources such as the recently published Food Research risK (FoRK) toolkit^(76)^ may provide a structured framework to navigate these issues. Furthermore, when declaring conflicts of interests, authors are encouraged to err on the side of full disclosure, thereby allowing readers to assess the potential effect of any disclosed conflicts on the research outcomes.

## Recommendations for future research

### Participant populations

There are a comparably large number of nutraceutical clinical trials that have been undertaken in participants with mental disorders such as major depressive disorder. In contrast, there are relatively few trials that have investigated these same nutraceutical interventions in other mental disorders. For example, a large meta-review found nine nutraceuticals where there was meta-analytic evidence for their use in major depressive disorder compared to only two in anxiety disorders^(7)^. This is also evident in the dietary intervention setting where there are a number of trials for mental disorders such as major depressive disorder and ADHD but few interventions for schizophrenia and bipolar disorder^(16,24,25,64)^. Similarly, given the relative lack of prevention studies in Nutritional Psychiatry, further trials are required to investigate both nutraceutical and dietary interventions in primary and secondary prevention of mental disorders (Table 8).

**Table 8.** Recommendations for future research Table 8 long description.

	Mean	sd	% rated ≥ 7·0
1. Consider diverse research settings, from acute inpatient care to clinics, to investigate treatment effects across diverse healthcare environments.	7·67	2·11	78
2. Further trials are required to explore under-researched populations (e.g. children and adolescents, older adults, indigenous cultures, non-binary, socially disadvantaged, remote and regional communities) and neuropsychiatric disorders (e.g. treatment resistant depression, schizophrenia, bipolar disorders, anxiety disorders).	8·78	1·47	89
3. Many emerging dietary regimens, like ketogenic or intermittent fasting diets, lack substantial clinical trial data and require further investigation.	7·72	1·76	78
4. Future trials should prioritise common elements of healthy diets, such as increased vegetable and fruit intake and reduced processed foods, rather than contrasting similar diets (e.g. DASH *v*. Mediterranean *v*. MIND diet).	7·06	2·09	61
5. Scalable models such as online and group-based interventions should be considered in future clinical trials. Group-based approaches may also leverage group dynamics to enhance peer support and motivation.	7·72	1·63	78
6. In addition to efficacy trials, consider real-world treatment effectiveness and implementation trial designs.	9·00	1·05	94
7. Explore psychosocial outcomes and potential treatment effect moderators, such as social or intuitive eating practices.	7·44	1·46	72

### Dietary interventions

Whole diet interventions in Nutritional Psychiatry have largely investigated Mediterranean-style diets. However, there are other diets with supporting preclinical or preliminary clinical data to support their use that require further investigation in rigorous, sufficiently powered trials. An example of this is the ketogenic diet, of which there are several case-reports, observational studies, and preclinical data suggesting a therapeutic effect in psychiatric conditions; however, there are currently no published RCT to confirm efficacy^(26)^. The same is true of other diet patterns that share principles similar to the Mediterranean diet that could have mental health utility in other cultural settings^(77)^. Other promising dietary approaches include high-polyphenol dietary interventions and dietary interventions that promote reduction in ultra-processed foods^(6,78)^.

### Scalable interventions and implementation research

While the trial evidence for the *efficacy* of nutraceutical and dietary interventions continues to grow, there is currently limited evidence regarding the *effectiveness* and implementation feasibility of nutraceutical and dietary interventions. We encourage further research that investigates the implementation of Nutritional Psychiatry into the real-world mental health care settings. These data will be invaluable in informing policy and will provide evidence-based models of how Nutritional Psychiatry can be integrated into clinical settings with the appropriate funding mechanisms to support implementation. Related to this is the need to investigate scalable approaches to dietary interventions. Online/telehealth delivery and group-based interventions are examples of scalable models of delivery that have facilitated the integration of other lifestyle interventions (e.g. exercise) into national clinical guidelines^(79)^ but workforce, education, and funding are issues that require consideration.

### Explore psychosocial outcomes

While much of the existing literature in Nutritional Psychiatry has focused on the potential biological mechanisms of dietary interventions to improve mental health, the possible psychosocial mechanisms have received less attention. Similar to exercise interventions, where the role of exercise in improving self-esteem, self-efficacy, and social support networks has been investigated as a potential psychosocial mechanism of action^(80)^, such investigations can be informative to future study designs and inform barriers and enablers to treatment response. Some studies have assessed factors such as self-efficacy and self-worth as secondary outcomes^(12,13)^; however, further research is required to assess these and additional psychosocial factors as potential drivers underlying clinical efficacy of diet and nutraceutical interventions.

## Conclusion

To assist continued research in the field of Nutritional Psychiatry, the ISNPR provides the first set of guidelines on clinical trial, design, conduct, and reporting for future clinical trials in this area. The sixty-one included recommendations cover several aspects of clinical trial design and reporting. The importance of a multidisciplinary research team and patient-centred input is a common theme. Recommendations also emphasise recognising and addressing unique biases in nutrition trials, improving the transparency and replicability of trial reporting, and calls for research into diverse populations and mental disorders. Recommendations included within these guidelines are intended to improve the rigor, impact, and clinical relevance of ongoing and future trials in Nutritional Psychiatry.

## Supporting information

Marx et al. supplementary material Marx et al. supplementary material

## Supplementary material

For supplementary material/s referred to in this article, please visit https://doi.org/10.1017/S0007114524001946

## Figure 1 Long description

The flowchart begins with the identification and invitation of investigators. The next step involves the submission of an initial set of recommendations by these investigators. These recommendations are then refined and recirculated. Investigators rate their agreement with each recommendation, which is then assessed. If a recommendation receives a rating below the threshold, it is excluded. In this case, 14 recommendations are below the threshold and are excluded. The final step includes 61 recommendations that meet the criteria and are incorporated into the guidelines.

Navigate back to Figure 1.

## Table 1 Long description

The table presents three trial team-related recommendation statements, each evaluated based on mean score, standard deviation, and percentage rated greater than or equal to 7. The first recommendation emphasizes the importance of diverse and proficient teams in trial design, statistics, and clinical practice, with a mean score of 8.56, standard deviation of 1.26, and 94 percentage rated greater than or equal to 7. The second recommendation highlights the engagement of individuals with lived experience of mental health disorders throughout all phases of the trial, with a mean score of 7.56, standard deviation of 2.61, and 72 percentage rated greater than or equal to 7. The third recommendation suggests that dietary interventions should be delivered by Accredited Practising Dietitians or Registered Dietitians, with a mean score of 7.17, standard deviation of 3.08, and 61 percentage rated greater than or equal to 7.

Navigate back to Table 1.

## Table 2 Long description

The table presents trial design-related recommendation statements, detailing their mean scores, standard deviations, and the percentage of ratings of 7.0 or higher. It includes eight recommendations, each with a mean score ranging from 7.89 to 9.33, standard deviations between 0.94 and 1.76, and percentages rated 7.0 or higher from 67% to 100%. Recommendations cover aspects such as participant burden, treatment adherence, dietary data collection, trial power, expectancy bias, blinding, trial duration, and seasonal variations. Each recommendation is designed to enhance the quality and reliability of clinical trials by addressing specific challenges and biases.

Navigate back to Table 2.

## Table 3 Long description

The table presents participant-related recommendation statements, detailing their mean scores, standard deviations, and the percentage of ratings that are 7 or higher. It includes seven rows, each representing a distinct recommendation. The columns are labeled 'Mean', 'SD', and 'Percentage rated greater than or equal to 7'. The recommendations cover various aspects such as recruitment strategies, managing comorbidities, handling eating disorders, considering baseline dietary practices, prioritizing high-risk participants in mental health interventions, focusing on populations with distinct clinical needs, and using structured interviews for mental disorder symptoms. Notable trends include high mean scores and percentages for recommendations related to recruitment strategies and managing comorbidities, indicating strong agreement on their importance.

Navigate back to Table 3.

## Table 4 Long description

The table presents intervention recommendation statements for dietary trials, detailing the mean ratings, standard deviations, and percentages rated 7 or higher. It includes ten recommendations, each with a mean rating, standard deviation, and percentage of raters giving a score of 7 or higher. Recommendations cover aspects such as determining the intervention type, manualising whole diet or behavioural interventions, ensuring dietary interventions involve qualified professionals, including comprehensive guidelines on food safety, tailoring interventions to population demographics, considering various modes of delivery, and focusing on ad libitum or weight-neutral designs. The table highlights the importance of these factors in designing effective dietary intervention trials.

Navigate back to Table 4.

## Table 5 Long description

The table presents data on six comparator recommendation statements, focusing on mean scores, standard deviations, and the percentage of ratings greater than or equal to 7. The table has six rows and three columns. The columns are labeled 'Mean', 'SD', and '% rated ≥ 7-0'. The rows list different statements about control conditions in trials. The first row discusses the justification for the choice of control condition, with a mean of 9.28, a standard deviation of 0.99, and 100 percentage rated greater than or equal to 7. The second row prioritizes the use of placebo or best-practice comparators over waitlist controls, with a mean of 7.56, a standard deviation of 2.59, and 72 percentage rated greater than or equal to 7. The third row addresses dietary interventions and the challenge of effective blinding, with a mean of 8.50, a standard deviation of 1.61, and 89 percentage rated greater than or equal to 7. The fourth row focuses on matching the placebo in appearance, taste, smell, and packaging for interventions involving nutraceuticals or foods, with a mean of 7.83, a standard deviation of 2.19, and 83 percentage rated greater than or equal to 7. The fifth row considers employing placebo diets and rigorously reviewing trial materials to sustain blinding, with a mean of 7.72, a standard deviation of 2.76, and 83 percentage rated greater than or equal to 7. The sixth row suggests using non-inferiority trial designs to compare Nutritional Psychiatry interventions to currently recommended treatments, with a mean of 7.67, a standard deviation of 2.60, and 78 percentage rated greater than or equal to 7.

Navigate back to Table 5.

## Table 6 Long description

The table presents outcome-related recommendation statements with corresponding mean, standard deviation, and percentage rated values. It includes 15 rows and 3 columns. The columns are labeled Mean, Standard Deviation (SD), and Percentage Rated Greater Than or Equal to 7. Each row provides a specific recommendation related to clinical outcomes, assessment methods, and data collection strategies. Notable recommendations include employing both self-report and clinician-rated methods for clinical outcomes, measuring symptoms at multiple timepoints, reporting total scores and individual assessment changes, assessing both psychiatric and physical outcome measures, measuring and reporting potential confounders, analyzing treatment effects in relevant subgroups, collecting biological samples, evaluating blinding success, using validated dietary assessment methods, including economic analyses, discussing trial-related burdens and costs, assessing acceptability and tolerability, conducting periodic safety assessments, and using validated assessment tools. The table highlights the importance of comprehensive and rigorous outcome measurement in clinical trials.

Navigate back to Table 6.

## Table 7 Long description

The table lists five reporting recommendation statements with corresponding mean, standard deviation, and percentage rated values. Each row details specific recommendations for dietary trials, dietary counseling, industry-funded trials, conflicts of interest, and trial protocols. The mean values range from 8.06 to 9.67, with standard deviations ranging from 0.58 to 2.37. The percentage rated values range from 78 to 100. The recommendations emphasize detailed descriptions, transparency, and public availability of trial protocols.

Navigate back to Table 7.

## Table 8 Long description

The table presents recommendations for future research in nutritional psychiatry, highlighting key areas for investigation. It includes seven rows and four columns, with columns labeled 'Mean', 'SD', and 'Percentage rated greater than or equal to 7 out of 10'. The rows detail specific recommendations such as considering diverse research settings, exploring under-researched populations, investigating emerging dietary regimens, prioritizing common elements of healthy diets, leveraging scalable models, focusing on real-world effectiveness, and exploring psychosocial outcomes. Each recommendation is accompanied by mean scores, standard deviations, and the percentage of ratings indicating high importance. Notable trends include high mean scores and percentages for recommendations related to under-researched populations and real-world effectiveness, indicating their critical importance in future research.

Navigate back to Table 8.
