# Supplementary material for: Methodological and reporting recommendations for clinical trials in Nutritional Psychiatry: Guidelines from the International Society for Nutritional Psychiatry Research
Source: Br J Nutr. 2024 Dec 10;135(6):608–20. doi: 10.1017/S0007114524001946 (PMC12269743; doi:10.1017/S0007114524001946)
Supplement: Marx et al. supplementary material [file S0007114524001946sup001.docx]

| **Table S1. Excluded Recommendations** |  |  |
| --- | --- | --- |
|  | Mean (SD) | % rated ≥7.0 |
| **Design-related recommendation statements** |  |  |
| Contemplate innovative designs suitable for smaller sample sizes (e.g. N of 1 trials, adaptive designs). | 5.56 (2.52) | 39 |
| Adopt an intervention run-in period post-recruitment and pre-randomization to optimize compliance and reduce drop out. | 5.89 (2.35) | 33 |
| Factor trauma history into recruitment processes and consider trauma-informed care in interventions. | 6.56 (2.71) | 61 |
| Consider examining the acute immediate effects of diet and nutraceuticals on clinical and biological indicators | 6.83 (1.54) | 56 |
| For interventions where the required duration for treatment effect is unclear, consider a minimum of 8 weeks | 6.39 (2.58) | 67 |
| **Participant-related recommendation statements** |  |  |
| Consider recruiting additional cohorts of healthy controls to discern biological changes resulting from interventions between clinically diagnosed participants and healthy individuals. | 5.89 (1.52) | 33 |
| Exclude individuals likely to be non-compliant with dietary interventions due to personal reasons like religious or ethical beliefs. | 6.56 (2.61) | 56 |
| **Intervention recommendation statements** |  |  |
| Pursue further research into the effect of individual food items or functional foods on mental health outcomes. | 5.44 (2.91) | 39 |
| For nutraceutical trials, administer interventions at the highest dose that can be safely administered to prevent underdosing and potential false negatives. If the optimal dose is uncertain, contemplate dose-response or dose-escalation study designs. | 6.94 (3.05) | 67 |
| Use nutraceutical formulations that deliver a standardized dose of the presumed active ingredient. | 6.83 (3.50) | 61 |
| **Outcome-related recommendation statements** |  |  |
| Incorporate functional measures to evaluate effects across multiple disorders. | 6.39 (2.81) | 61 |
| Measures of self-efficacy should be included in all dietary trials | 5.61 (2.09) | 33 |
| **Reporting recommendation statements** |  |  |
| Opt for publication in psychiatric journals over nutrition-specific journals to broaden accessibility to clinicians. | 5.78 (2.80) | 39 |
| **Recommendations for future research** |  |  |
| Explore the potential of AI tools, such as apps suggesting diet recipes, to augment research in nutritional psychiatry. | 6.67 (1.97) | 61 |

**Questionnaires sent to guidelines researchers**

Thank you for participating in this Delphi survey.

The aim of this project, led by Heidi Staudacher and myself, is to develop a series of best practice recommendations and research priorities to be addressed in future **clinical trials** (rather than other study designs) in Nutritional Psychiatry.

These recommendations will be formed based on a Delphi survey process.

In this initial survey, we ask that you submit recommendations, based on your experience and expertise in clinical trials of whole-diet, food and nutraceutical supplementation or probiotics and other ‘iotics’. These can cover any part of the clinical trial process, from planning and design (e.g. participants, intervention) to study conduct. We have included examples of such recommendations below under some of these broad classifications.

Please be as specific as possible – for example, if recommendations are made about the need for novel interventions, then please provide a list of interventions that you propose to be novel in this field.

We also welcome supporting references for any recommendations provided as this will help the writeup of the manuscript.

Once we have received these, we will collate all recommendations and distribute a refined list of recommendations that we will ask you to score based your agreement with each statement (further instructions to follow). This process will be anonymous with no names attached to individual recommendations.

If you have any queries about this process or have additional recommendations you would like to submit post-survey, please feel free to contact us at [wolf.marx@deakin.edu.au](mailto:wolf.marx@deakin.edu.au)

**Trial planning and design considerations**

Areas may include:

- Trial design features and methods (e.g. length of trials, sample sizes)
- Team composition (e.g. people with lived experience, allied health workers)

| - *Example: Investigators should consider the inclusion of people with lived experience in all aspects of trial design and conduct* |
| --- |

**Participant considerations**

Areas may include:

- Recruitment considerations
- Inclusion/exclusion criteria
- Under-researched populations in nutritional psychiatry (e.g. clinical populations, age, gender, race/ethnicity)

| - *Example: Most dietary trials have focused on depression. Clinical populations other than major depressive disorder (e.g. anxiety, schizophrenia) require further investigation* |
| --- |

**Intervention considerations**

Areas may include:

- Composition of diet/nutraceutical interventions
- The mode of delivery
- Duration of the intervention
- Separating contextual effects (e.g. motivational counselling in whole diet trials) from intervention effects
- Supporting resources (e.g. written information, diet hampers)
- Under-researched and/or emerging interventions in nutritional psychiatry

| - *Example: Other dietary approaches besides the Mediterranean diet should be explored in future clinical trials* |
| --- |

**Comparator/control considerations**

Areas may include:

- Methods to address expectancy bias
- Methods to optimise blinding
- Reporting blinding success
- Reporting design and delivery of controls

| - *Example: The choice of control should be clearly explained, and where appropriate the composition should be clearly documented* |
| --- |

**Outcome considerations**

Areas may include:

- Preferred assessment tools
- Important primary outcomes
- Important secondary outcomes
- Confounders
- Best practice in assessing outcomes
- Under-researched outcomes in nutritional psychiatry
- Cost analysis
- Biomarker considerations
- Dietary assessment considerations
- Recommended analyses/sensitivity analyses

| - *Example: Relying on self-reported assessment tools may introduce bias in unblinded dietary interventions. Clinicians rated assessment tools may allow for single-blinded study designs and reduce risk of bias.* |
| --- |

**Other considerations**

Areas may include

- Reporting considerations
- Methods to reduce specific forms of bias
- Anything not covered in the above sections

| - *Example: Further effectiveness (rather than efficacy) and implementation research is required to assess outcomes in real-world clinical practice* |
| --- |

**Sample Likert scale used for second round of feedback**

| Sample question | No answer | Strongly disagree | 2 | 3 | 4 | Neither Agree or disagree | 6 | 7 | 8 | 9 | Strongly agree |
| --- | --- | --- | --- | --- | --- | --- | --- | --- | --- | --- | --- |
| Teams should comprise investigators proficient in all relevant trial aspects, such as trial design, statistics, and clinical practice, and should comprise ethnic and gender diversity. |  |  |  |  |  |  |  |  |  |  |  |
